# DBS-evoked cortical responses index optimal contact orientations and motor outcomes in Parkinson’s disease

**DOI:** 10.1038/s41531-023-00474-4

**Published:** 2023-03-11

**Authors:** Rachel K. Spooner, Bahne H. Bahners, Alfons Schnitzler, Esther Florin

**Affiliations:** 1 Institute of Clinical Neuroscience and Medical Psychology, Medical Faculty, Heinrich-Heine University, Düsseldorf, Germany; 2 Department of Neurology, Center for Movement Disorders and Neuromodulation, Medical Faculty, Heinrich-Heine University, Düsseldorf, Germany

**Keywords:** Parkinson's disease, Basal ganglia

## Abstract

Although subthalamic deep brain stimulation (DBS) is a highly-effective treatment for alleviating motor dysfunction in patients with Parkinson’s disease (PD), clinicians currently lack reliable neurophysiological correlates of clinical outcomes for optimizing DBS parameter settings, which may contribute to treatment inefficacies. One parameter that could aid DBS efficacy is the orientation of current administered, albeit the precise mechanisms underlying optimal contact orientations and associated clinical benefits are not well understood. Herein, 24 PD patients received monopolar stimulation of the left STN during magnetoencephalography and standardized movement protocols to interrogate the directional specificity of STN-DBS current administration on accelerometer metrics of fine hand movements. Our findings demonstrate that optimal contact orientations elicit larger DBS-evoked cortical responses in the ipsilateral sensorimotor cortex, and importantly, are differentially predictive of smoother movement profiles in a contact-dependent manner. Moreover, we summarize traditional evaluations of clinical efficacy (e.g., therapeutic windows, side effects) for a comprehensive review of optimal/non-optimal STN-DBS contact settings. Together, these data suggest that DBS-evoked cortical responses and quantitative movement outcomes may provide clinical insight for characterizing the optimal DBS parameters necessary for alleviating motor symptoms in patients with PD in the future.

## Introduction

Parkinson’s disease (PD) is a progressive neurodegenerative disorder with the most common symptoms manifesting as debilitating motor impairments that ultimately lead to functional dependencies in later life^1^. Although subthalamic deep brain stimulation (STN-DBS^2,3^ is a highly-effective treatment to combat motor symptoms in patients with advanced PD, some individuals are still left without experiencing optimal clinical benefits, and reliable neurophysiological and behavioral correlates of optimal parameter settings are lacking. This is unfortunate, as such data may be imperative for augmenting the treatment efficacy of STN-DBS, while simultaneously allowing for more efficient programming appointments that are currently restricted to subjective designations of therapeutic effects (e.g., monopolar review sessions). Thus, the implementation of individualized stimulation parameters, and understanding the underlying neural and behavioral correlates leading to optimized clinical outcomes is of utmost importance.

One proposed parameter that may augment the efficacy of STN-DBS is the direction of which current is administered through the device (i.e., contact orientations)^4–7^. Conventional DBS systems using ring-shaped electrode contacts only allowed for an omni-directional, i.e., axially symmetrical current distribution throughout the entirety of the electrode at a fixed location within the STN. However, this approach has the potential for current spread into neighboring subcortical structures eliciting side effects based on resulting stimulation topography^4,7^. Newer devices with segmented electrode contacts allow for directional current administration (e.g., anterior, medial, lateral) which may ameliorate the unwanted spread of current to neighboring structures, effectively increasing the therapeutic window (i.e., minimal stimulation amplitude required to elicit clinical benefits vs. side effects) based on the increased spatial precision^5,8–11^. However, despite this advancement, clinical programming of STN-DBS still relies on lengthy monopolar review sessions, as the neurophysiological mechanisms underlying disparate clinical outcomes induced by clinically-effective and non-effective contact orientations for STN-DBS programming are not well understood.

In regard to mechanism, one potential marker that may be sensitive to changes in contact orientations and further, its clinical efficacy, are stimulation-evoked cortical responses in the brain. Previous studies have primarily used electrocorticography (ECoG) or electroencephalography (EEG) over sensorimotor regions and have shown the presence of short-, medium- and long-latency responses evoked by DBS that may be reflective of corticospinal tract, hyperdirect pathway, and orthodromic polysynaptic activation of the basal ganglia-thalamo-cortical loop, respectively^12–17^. Interestingly, these studies suggest that DBS-evoked cortical responses are largest when undergoing clinically-effective stimulation settings. For example, Miocinovic et al. observed that DBS-evoked cortical responses in motor and premotor areas were larger when undergoing higher stimulation amplitudes, longer pulse widths, and when a clinically-effective contact was applied^13^. While this study provided valuable insight regarding the sensitivity of cortical evoked responses to DBS programming settings in PD patients, these responses were recorded using ECoG, which may have limited utility in larger populations due to the invasive nature of the device and its spatial limitation to the areas directly beneath the implanted ECoG strip. One approach that may expand upon these limitations is the use of magnetoencephalography (MEG), which is a non-invasive neurophysiological recording technique with whole-head coverage, which affords good spatial precision, concomitant with an excellent temporal resolution (<1 ms)^18,19^. Moreover, MEG has demonstrated success in resolving STN-DBS-evoked cortical responses in movement disorder patients^20^. Importantly, regardless of the chosen neuroimaging technique, the systematic evaluation of optimal versus non-optimal programming settings (i.e., contact orientations) on DBS-evoked cortical responses and further, their potential for indexing quantitative clinical outcomes of motor symptomology has yet to be investigated.

Thus, the goal of the current study was to systematically evaluate the potential neural and behavioral correlates of clinical outcomes relating to clinically-effective (i.e., optimal) and non-optimal contact orientations for STN-DBS programming in patients with PD. Importantly, this study expands upon prior relevant studies in this area by evaluating each directional *and* omni-directional contact configuration in concert, in order to discern patient-specific variations in clinical outcomes induced by the direction of the administered current. To this end, we recruited 24 patients with PD with STN-DBS who completed a monopolar stimulation paradigm of the left STN during MEG to directly quantify neural dynamics evoked by STN-DBS as a function of disparate contact orientations. In addition, patients completed standardized movement protocols (i.e., Unified Parkinson’s Disease Rating Scale III: UPDRS III) with a triaxial accelerometer affixed to their right index finger to quantitatively assess hand motor function during optimal and non-optimal contact orientations tested. Using linear mixed effects models (LME), we tested the hypothesis that clinically-effective contact orientations would elicit larger sensorimotor evoked responses and further, better behavioral performance during finger tapping paradigms compared to non-optimal contact settings. In addition, we hypothesized that larger sensorimotor responses evoked by STN-DBS would be predictive of behavioral performance in a contact-dependent manner across the sample.

## Results

Of the 24 patients enrolled in the current study, 3 patients were unable to successfully complete the MEG and behavioral aspects of the study in the medication OFF state, while 1 patient was excluded from the final analyses due to excessive artifacts in the MEG data. The remaining 20 patients had a mean age of 64.1 years (2 females; Table 1).

**Table 1:** Patient demographics and clinical information for final analyzed cohort.

Demographics (Mean ± SD)	
*N*	20
Age (years)	64.1 ± 9.1
Sex (% males)	90
Time since diagnosis (years)	12.6 ± 6.2
Time since DBS implantation (years)	3.1 ± 1.4
Symptom subtype (% akinetic-dominant)	90
Clinically-effective contact (% directional)	60
UPDRS-III Med OFF, Stim OFF	36.5 ± 13.7
UPDRS-III Med OFF, Stim ON	23.5 ± 11.0

Symptom subtype was computed using a ratio score of mean tremor sub-scores (items 3.15, 3.16, 3.17, 3.18) to mean bradykinesia sub-scores (items 3.4, 3.5, 3.6, 3.7, 3.8, 3.14) from the UPDRS-III examination. If zero was in the numerator, then the patient would be classified as akinetic-dominant. In contrast, if zero was in the denominator, then the patient would be classified as tremor-dominant. Ratio scores greater than 1.5 indicate tremor-dominant subtypes.

### Contact-dependent changes in finger tapping performance

**Fig. 1:**
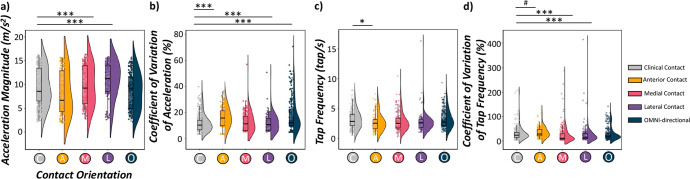
STN-DBS contact orientations effect accelerometer metrics of finger tapping. Single-trial accelerometer metrics of finger tapping (e.g., acceleration magnitude, tap frequency, variability) were quantified using a fixed-threshold algorithm and subjected to LMEs of each behavioral metric as a function of contact orientation (factor with 5 levels), controlling for acquisition order. Raincloud plots include a combined box plot (box edges: first 25th percentile quartile to third 75th percentile quartile; center line: median; data minima/maxima: whisker length), histogram distribution and individual scatter points of each accelerometer metric. Right-handed finger tapping yielded smoother movement profiles (i.e., slower single-trial general acceleration (**a**), lower acceleration variability per tap (**b**), increased tapping frequency (**c**)) as well as increased variability in tap frequency (**d**) during clinically-effective contact orientations. Significance is shown for post-hoc testing comparing optimal vs. all other non-optimal contact orientations at ^#^*p*_corrected_ < 0.10, *p_corrected_ < 0.05, **p_corrected_ < 0.01, ***p_corrected_ < 0.005.

In order to interrogate the behavioral impact of STN-DBS at optimal and non-optimal contact orientations, we examined single-trial accelerometer metrics of finger tapping (i.e., general acceleration magnitude, tapping frequency and the coefficient of variation in each metric) separately using linear mixed effects models of contact orientation as a fixed effect, controlling for acquisition order where appropriate, and with subject and trial number as a nested random effect (Fig. 1 and Supplementary Tables 2–5).

In regard to tap acceleration, our results indicated a significant main effect of contact orientation controlling for acquisition order (*F*(1112) = 9.58, *p* < 0.001, Cohen’s *d* = 0.19, 95% CI [0.07, 0.31]; Fig. 1 and Supplementary Tables 2–5). Post-hoc testing suggested that single-trial acceleration magnitude was slower when clinically-effective contact orientations were applied compared to non-optimal medial contacts (*t*(1112) = −3.84, *p* < 0.001, Cohen’s *d* = −0.12, 95% CI [−0.17, −0.06]) and lateral contacts (*t*(1112) = −4.82, *p* < 0.001, Cohen’s *d* = −0.14, 95% CI [−0.20, −0.09]), but not compared to anterior or omni-directional contact orientations (*ps* > 0.102). Interestingly, this general reduction in tap acceleration during clinically-effective contact orientation DBS was concomitant with the lowest variability in tap acceleration (i.e., lower coefficient of variation controlling for acquisition order; *F*(1117) = 19.62, *p* < 0.001, Cohen’s *d* = 0.27, 95% CI [0.15, 0.38]), reflecting greater consistency of tapping acceleration magnitudes over the entire tapping block compared to non-optimal anterior contacts (*t*(1116) = −4.70, *p* < 0.001, Cohen’s *d* = −0.14, 95% CI [−0.20, −0.08]), lateral contacts (*t*(1117) = −2.08, *p* = 0.038, Cohen’s *d* = −0.06, 95% CI [−0.12, 0.0]) and omni-directional contacts (*t*(1118) = −6.06, *p* < 0.001, Cohen’s *d* = −0.18, 95% CI [−0.24, −0.12]).

In regard to tapping frequency, our results indicated a significant main effect of contact orientation controlling for acquisition order (*F*(1067) = 2.42, *p* = 0.047, Cohen’s *d* = 0.14, 95% CI [0.02, 0.26]; Fig. 1). Post-hoc testing suggested that single-trial tapping frequency was increased for clinically-effective contact orientations compared to non-optimal anterior contacts (*t*(1066) = 2.03, *p* = 0.042, Cohen’s *d* = 0.06, 95% CI [0.0, 0.12]), but not compared to medial, lateral or omni-directional stimulation (*p* > 0.355). Finally, the variability in tapping frequency (i.e., coefficient of variation in inter-tap intervals) significantly differed as a function of contact orientation when controlling for acquisition order (*F*(1067) = 5.32, *p* < 0.001, Cohen’s *d* = 0.14, 95% CI [0.02, 0.26]), with clinically-effective stimulation eliciting marginally decreased variation in tapping frequency compared to non-optimal anterior contacts (*t*(1066) = −1.71, *p* = 0.087, Cohen’s *d* = 0.05, 95% CI [−0.01, 0.11]), and increased variability compared to medial (*t*(1064) = 3.89, *p* < 0.001, Cohen’s *d* = 0.12, 95% CI [0.06, 0.18]) and lateral contacts (*t*(1067) = 2.95, *p* = 0.003, Cohen’s *d* = 0.09, 95% CI [0.03, 0.15]).

### Neural responses to monopolar STN-DBS

**Fig. 2:**
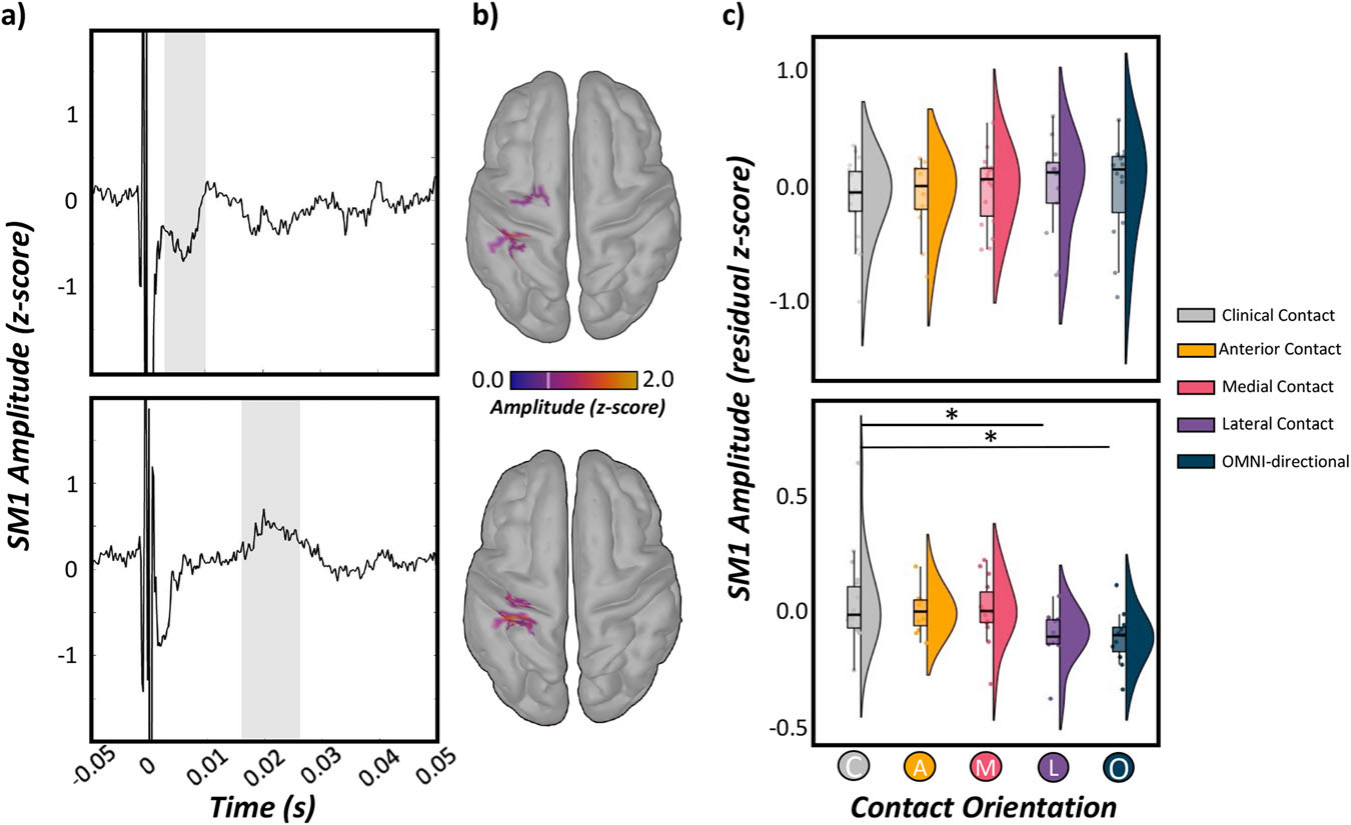
Neural responses evoked by STN-DBS during different contact orientations. **a** Source reconstruction of DBS-evoked cortical responses was conducted using unconstrained weighted minimum norm estimation and revealed robust medium- and long-latency sensorimotor (SM1) responses ipsilateral to the site of stimulation from 3–10 ms (shaded gray bar; top panel) and 16–26 ms (shaded gray bar; bottom panel) following DBS pulses denoted at time 0 ms, respectively. Time-domain average of the extracted SM1 response (i.e., dominant orientation time series) with time (in ms) on the *x*-axis and response amplitude (*z*-score) on the *y*-axis. All axes are fixed for each graph. **b** Grand averaged cortical patterns of medium- (top panel) and long-latency (bottom panel) responses in the ipsilateral SM1 across all patients, trials and contact orientations. **c** LME of medium- and long-latency SM1 response amplitude (i.e., <10 ms and ~20 ms, respectively) as a function of contact orientation (factor with 5 levels), controlling for acquisition order and total electrical energy delivered revealed stronger SM1 long-latency responses when clinically-effective contact directions were applied, while medium-latency SM1 responses were unchanged. Raincloud plots include a combined box plot (box edges: first 25th percentile quartile to third 75th percentile quartile; center line: median; data minima/maxima: whisker length), histogram distribution and individual scatter points of each evoked response. Significance is shown for post-hoc testing comparing optimal vs. all other non-optimal contact orientations at *p_corrected_ < 0.05.

Significant medium- and longer-latency evoked neural responses were found in many sensors near the ipsilateral sensorimotor (SM1) regions from 3–10 ms and 16–26 ms after the onset of the DBS pulse (*p* < 0.005, FDR-corrected; Fig. 2), respectively. Of note, due to remnants of the DBS artifact, we did not evaluate evoked response dynamics at shorter latencies (<2 ms). To evaluate the anatomical origin of these responses, cortical sources were imaged using unconstrained weighted MNE. MNE images revealed robust absolute increases in the ipsilateral SM1 (i.e., pre- and postcentral gyri) in response to 6 Hz monopolar DBS of the left STN. As described in the methods, these images were grand-averaged across all trials, patients and experimental runs to extract time series from the region of interest for subsequent multilevel models of contact orientation on evoked data (Fig. 2 and Supplementary Tables 6–9). Next, we evaluated the influence of optimal and non-optimal contact orientations on SM1 evoked response amplitudes and latencies using linear mixed effects models. In regard to medium-latency SM1 responses (<10 ms), we observed no main effect of contact orientation on residual SM1 response amplitude controlling for acquisition order and total electrical energy delivered (TEED) (*F*(78) = 0.28, *p* = 0.892, Cohen’s *d* = 0.12, 95% CI [−0.32, 0.56]). In addition, we observed no effect of contact orientation on evoked response peak latencies during the 3–10 ms window following DBS pulse onset (*F*(62) = 0.49, *p* = 0.744, Cohen’s *d* = 0.18, 95% CI [−0.32, 0.68]). In contrast, regarding longer-latency SM1 responses (i.e., 16–26 ms), our results indicated a trending main effect of contact orientation on residual SM1 response amplitude (controlling for acquisition order and TEED; *F*(69) = 2.40, *p* = 0.058, Cohen’s *d* = 0.37, 95% CI [−0.10, 0.85]), such that SM1 responses were larger during clinically-effective contact orientation administration compared to lateral contacts (*t*(69) = 2.48, *p* = 0.016, Cohen’s *d* = 0.30, 95% CI [0.06, 0.54]) and omni-directional stimulation (*t*(69) = 2.55, *p* = 0.013, Cohen’s *d* = 0.31, 95% CI [0.06, 0.55]), but not compared to anterior or medially-oriented contacts (*ps* > 0.279; Fig. 2). In contrast, we observed no effect of contact orientation on SM1 evoked response peak latencies (*F*(60) = 1.30, *p* = 0.279, Cohen’s *d* = 0.30, 95% CI [−0.22, 0.82]). Of note, as we did not observe contact-dependent changes in SM1 medium-latency response dynamics (i.e., amplitude/latency), as well as longer-latency SM1 peak response latencies, these metrics were not included in subsequent analyses of brain-behavioral relationships as a function of contact orientation. As a result, we report absolute time series data (i.e., normed across the three orientations) for long-latency SM1 responses to facilitate interpretation.

### SM1 evoked responses differentially predict movement profiles in a contact-dependent manner

**Fig. 3:**
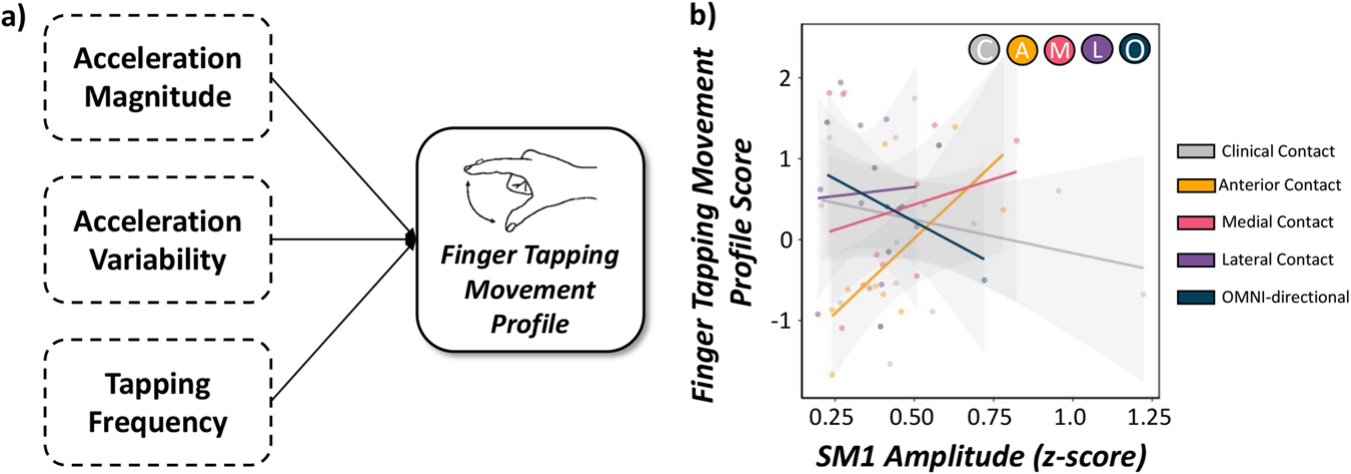
Finger tapping movement profiles are predicted by SM1 evoked cortical responses in a contact-dependent manner. **a** Conceptual figure denoting the statistical model probed in the current study to derive data-driven accelerometer-based movement profiles of finger tapping. The factors contributing to finger tapping movement profiles were obtained from an EFA including acceleration magnitude, acceleration variability (i.e., coefficient of variation) and reverse-coded tapping frequency with lower values of finger tapping movement profiles reflective of smoother movements during finger tapping blocks. **b** LME of finger tapping movement profiles scores as a function of SM1 response amplitude (continuous variable), contact orientation (factor with 5 levels) and their interaction were conducted controlling for acquisition order and total electrical energy delivered. There was a significant SM1 response by contact orientation interaction such that when clinically-effective contact orientations were applied, larger SM1 response amplitudes were predictive of smoother finger tapping movement profiles. 95% confidence intervals are displayed in gray for each regression line.

Next, we aimed to evaluate the predictive capacity of longer-latency SM1 evoked cortical responses, contact orientations and their interaction on empirically-derived, sample-specific movement profiles in our patients. As described in the methods, we constructed a finger tapping movement profile score using an EFA of a compilation of accelerometer metrics exhibiting significant alterations as a function of contact orientation (i.e., acceleration magnitude, acceleration variability, tap frequency, tap frequency variability). The initial EFA based on all four accelerometer metrics indicated a single-factor solution with poor fit (*χ*^2^ = 25.19; RMSEA = 0.27, 90% CI [0.14, 0.42]; CFI = 0.89; SRMR = 0.07). Since tap frequency variability (i.e., coefficient of variation) loaded poorly onto the factor (*λ* = −0.64), excluding this variable yielded a single-factor solution with excellent model fit (*χ*^2^ = 16.75; RMSEA = 0.00, 90% CI [0.00, 0.00]; CFI = 1.00; SRMR = 0.00). Thus, our empirically-defined sample-specific quantification of finger tapping movement profiles was comprised of acceleration magnitude, acceleration variability and reverse-coded tap frequency, which accounted for 70.5% of the variance in finger tapping movement profiles. Of note, lower movement profile scores are reflective of smoother movements which was used as a dependent variable in our LME controlling for acquisition order and TEED (for achieved loadings and conceptual figure of our statistical model, see Table 2 and Fig. 3, respectively). Similar to our single-trial accelerometer results, we observed a significant main effect of contact orientation on finger tapping movement profile scores (*F*(32) = 5.03, *p* = 0.002, Cohen’s *d* = 0.78, 95% CI [0.07, 1.48]). In regard to DBS-evoked responses, there was no main effect of long-latency SM1 response amplitude on movement profile scores (*p* = 0.622), albeit we did observe a significant long-latency SM1 response amplitude by contact orientation interaction (*F*(33) = 4.35, *p* = 0.006, Cohen’s *d* = 0.73, 95% CI [0.02, 1.43]; Fig. 3 and Supplementary Table 10). Post-hoc analyses revealed that larger evoked responses in SM1 were predictive of lower movement profile scores (i.e., smoother finger tapping) during optimal contact orientation administration compared to anteriorly-oriented contacts showing the opposite trajectory (*Z* = 2.35, *p* = 0.018, Cohen’s *r* = −0.56, 95% CI [−0.78, −0.10]), but not compared to medial, lateral or omni-directional contacts (*ps* > 0.424).

**Table 2:** Achieved loadings derived from EFA.

Finger tapping movement profiles			
Metric	Achieved loadings	Eigenvalue	Variance accounted for
Acceleration magnitude	0.918	2.12	70.5%
Acceleration variability	0.852		
Tapping frequency	0.739		

Finger tapping accelerometer metrics exhibiting significant alterations as a function of contact direction were subjected to EFA to derive a single component reflective of fine hand movement profiles. Acceleration magnitude (in m/s^2^), acceleration variability (%) and reverse-coded tapping frequency (in taps/s) accounted for 70.5% of the variance in finger tapping movement profiles.

### Contact-dependent changes in therapeutic effects

**Fig. 4:**
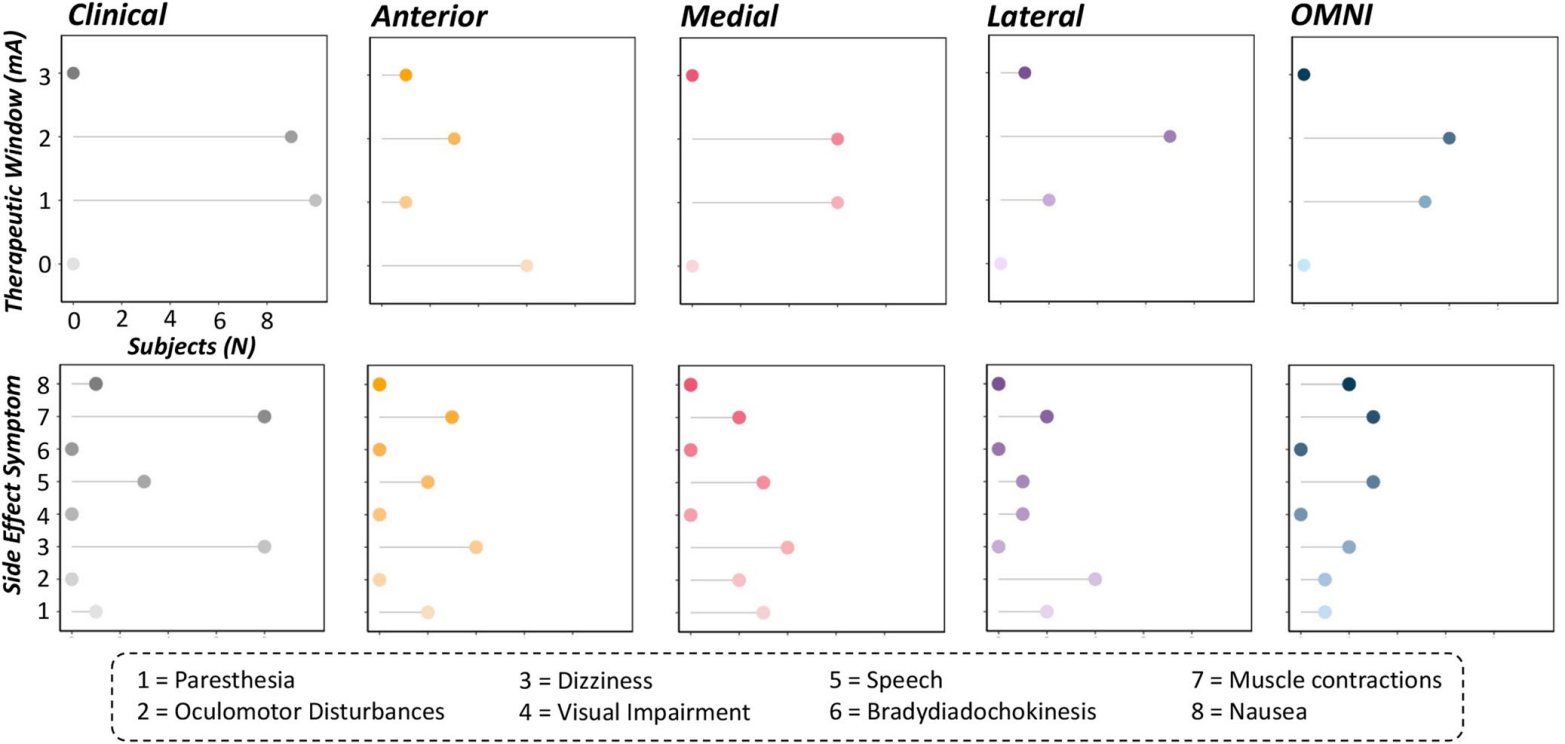
Observed therapeutic windows and side effects for optimal and non-optimal current administration. Lollipop charts denoting therapeutic windows (i.e., minimal distance in mA from observed clinical effect to observed side effect; *y*-axis in top panel) observed when undergoing different contact orientations of STN-DBS and reported side effects (*y*-axis in bottom panel). The *x*-axis represents the number of subjects (*N*) and is fixed for all charts. Reported side effects included paresthesia (1), oculomotor disturbances (2), dizziness (3), visual impairment (4), speech disturbances (5), bradydiadochokinesis (6), muscle contractions (7) and nausea (8).

Finally, we aimed to qualitatively evaluate the therapeutic windows and observed side effects induced by each optimal and non-optimal contact orientation in our sample to gleam further insights regarding optimal programming settings. Patient-reported side effects included paresthesia (1), oculomotor disturbances (2), dizziness (3), visual impairment (4), speech disturbances (5), bradydiadochokinesis (6), muscle contractions (7) and nausea (8) (Fig. 4). Of the 20 patients who completed simultaneous STN-DBS and MEG/behavioral aspects of the study at varying contact administrations, we observed 64 reports of side effects, with muscle contractions (25.0%), dizziness (34.4%) and speech disturbances (18.8%) being the most commonly reported regardless of the current administration protocol. Interestingly, these were also the most common side effects reported for clinically-effective (i.e., optimal) contact orientations, non-optimal medially-oriented contacts, as well as omni-directional contacts, each of which was concomitant with greater therapeutic windows (100% of contacts demonstrated 1–2 mA therapeutic window). In contrast, anteriorly-oriented, non-optimal contacts elicited dizziness (36%) and paresthesia (18%) in addition to muscle contractions (27%) and speech disturbances (18%). Importantly, these side effects were concomitant with the lowest therapeutic windows (54% of anterior contacts with 0 mA therapeutic window). Finally, laterally-oriented non-optimal contacts elicited the greatest proportion of oculomotor disturbances (e.g., diplopia or double vision and gaze deviations in 40% of lateral contact side effects) in our sample, albeit larger therapeutic windows were maintained during this stimulation configuration (100% of lateral contacts with 1–3 mA therapeutic window).

## Discussion

In the current study, we used advanced neurophysiology and quantitative sensor-based approaches to determine the predictive capacity of DBS-evoked cortical responses on fine hand movement kinematics during optimal and non-optimal programming settings (i.e., contact orientations) in a cohort of patients with PD. Specifically, we observed (1) that DBS-evoked cortical responses in the ipsilateral sensorimotor cortex (SM1) at longer latencies were larger when clinically-effective contact orientations were applied, (2) that single-trial finger tapping movement profiles were smoother during optimal configurations, and (3) that the two were differentially linked in a contact-dependent manner. Moreover, we observed that reported side effects were consistent with the anatomical direction of current administration and that this was concomitant with lower therapeutic windows during non-optimal stimulation paradigms in our sample. Below, we discuss the implications of these findings for understanding the contribution of DBS-evoked cortical responses and quantitative movement kinematics for indexing optimal DBS programming settings in PD.

Our findings suggesting that DBS-evoked cortical responses were sensitive to changes in contact orientation were not surprising, as prior studies have established this relationship in the context of clinical efficacy determined from traditional monopolar reviews^13,21,22^. Essentially, these studies have shown that evoked potentials are largest in motor and premotor cortices for clinically-effective stimulation contacts, and further, contacts that were also oriented toward the dorsolateral STN^13,15,21^. Interestingly, the majority of our sample (~70%) had their pre-defined clinically-effective contact orientation as lateral or omni-directional, which is consistent with prior reports noting potential improvement of clinical outcomes (e.g., UPDRS symptom severity) based on this stimulation topography^13,15,21,23^. In addition, we expanded upon prior work to systematically evaluate cortical responses evoked by contacts at each directional and omni-directional orientation about the DBS electrode at the clinically-effective fixed contact height. Specifically, we found that longer-latency SM1 evoked responses were largest when undergoing clinically-effective contact orientations compared to non-optimal laterally- or omni-directional oriented contacts. Importantly, all analyses controlled for TEED^24^, ensuring that the contact-dependent changes observed in evoked response amplitude were not confounded by ring-shaped vs. directional current administration that likely results in discrepancies in the volume of tissue activated for either approach^4,25,26^.

Our most important finding established a link between DBS-evoked responses and quantitative finger tapping movement profiles in patients with PD. Using factor analyses to construct a latent movement profile score which included a compilation of accelerometer metrics reflecting changes as a function of contact orientation, we observed that larger SM1 evoked responses were significant predictors of smoother finger tapping movements (i.e., lower acceleration magnitude, lower acceleration variability, increased tapping frequency) during clinically-effective contact stimulation protocols. While the observation of lower acceleration magnitudes during clinically-effective protocols may be unintuitive at first glance, it is important to consider this directionality within the context of other important kinematic features quantified during the finger tapping paradigm. As this metric reflects the acceleration required to execute each index-to-thumb flexion-extension tap in the consecutive sequence, which was also most consistent during clinically-effective settings (i.e., coincident with lower acceleration variability), these data likely reflect more controlled movement profiles induced by clinically-effective contact regimens. This hypothesis is further underscored by the finding of increased tap frequency during optimal contact orientations, which suggests that patients were able to maintain a relatively fast tapping pace (i.e., frequency) over the entire tapping block despite lower intra-tap acceleration to execute each flexion-extension movement. In regard to the directionality of these brain-behavior dynamics, there were significant differences in brain-behavior relationships between clinically-effective contact stimulation compared to anteriorly-oriented directional stimulation paradigms, which elicited the opposite trajectory. While previous studies have done well to characterize changes in motor performance as a function of contact location/orientation using standard clinical evaluations (e.g., UPDRS improvement, monopolar review of the therapeutic effect)^10,13,27,28^, the current study demonstrated that quantitative measures of standardized movements (i.e., single-trial UPDRS finger tapping dynamics) were not only sufficient to effectively index optimal versus non-optimal DBS contact orientations but also, were differentially impacted by longer-latency SM1 cortical responses evoked by STN-DBS. Taken together, these data suggest that DBS-evoked cortical responses, quantitative movement kinematics, and importantly, their interaction, may serve as effective targets for identifying optimal DBS programming strategies for patients with PD.

In regard to mechanism, the cortical activation observed herein is likely the result of orthodromic polysynaptic activation of the basal ganglia-thalamo-cortical loop as opposed to other mechanisms evoked by DBS. For example, previous studies have demonstrated a stimulation topography at shorter- (<2 ms), medium- (~2–10 ms) and longer-latencies (>10 ms) in a distributed cortical network in patients with PD as a result of STN-DBS. Essentially, shorter and medium-latency responses are characterized by their conduction speeds and topography from motor to frontal areas for corticospinal tract and hyperdirect pathway activation, respectively^12,13,17,22^. In contrast, longer-latency responses may reflect orthodromic activation of the subcortical-cortical loop, with similar evolution of cortical topographies observed regardless of the chosen DBS site (i.e., STN or pallidal DBS)^13,15,22^. Although prior work has provided strong evidence for hyperdirect pathway activation driving therapeutic effects observed during STN-DBS^13,29–34^, our study suggests that longer-latency responses (~16–26 ms following the DBS pulse) are also pertinent to modulating clinical outcomes (i.e., smoother finger tapping movement profiles in a contact-dependent manner). Moreover, our study demonstrated that longer-latency responses occurring ~20 ms following DBS pulses were differentially modulated by optimal/non-optimal contact orientations, as opposed to earlier stimulation topographies (<10 ms from DBS pulse). Nevertheless, it will be imperative for future work to evaluate the disparate contributions of stimulation-evoked cortical responses on these dynamic movement profiles in concert to fully unravel this mechanism, as we were unable to resolve earlier cortical patterns due to DBS-related artifacts in the MEG signal (<2 ms following DBS pulses)^35^.

Finally, our study concurrently reports DBS-induced therapeutic windows and associated side effects as a function of optimal and non-optimal contact settings tested in our sample. Across all patients and contact orientations, the most common side effects included muscle contractions, speech disturbances and dizziness. However, non-optimal stimulation protocols induced other side effects based on the direction of which current was administered. For example, laterally-oriented, non-optimal contacts induced the majority of oculomotor disturbances observed in the current sample (i.e., diplopia or double vision and gaze deviations), which may be induced by simultaneous stimulation of the internal capsule or the oculomotor nerve, respectively^4,5,36–38^. In contrast, anteriorly-oriented, non-optimal contacts induced more autonomic changes in patients such as dizziness possibly due to unwanted current spread to hypothalamic areas^4,5^, albeit this side effect appears to be more ubiquitous across patients regardless of stimulation site (e.g., STN vs. palladial vs. thalamic) or contact orientation (e.g., anterior, medial, lateral)^4,8^. In addition, anteriorly-oriented contacts induced more instances of paresthesias in patients. While interesting, this side effect is typically reflective of current spread to more posteriorly-oriented locations including the medial lemniscus^4,8^. Thus, future work is necessary to corroborate the anatomico-clinical correlation of anteriorly-oriented contacts and the presence of paresthesias in DBS patients with PD. Nevertheless, non-optimal anteriorly-oriented contact stimulation also resulted in the lowest therapeutic windows, with ~54% of our sample experiencing side effects at the same stimulation amplitude for which they experienced some therapeutic benefit (i.e., 0 mA therapeutic window). In regard to the current sample, these data suggest that anteriorly-oriented contacts may not be as beneficial for simultaneously eliciting clinical benefits, while also reducing stimulation-induced side effects compared to other stimulation configurations. This observation was not surprising and aligned well with our neurophysiological and behavioral findings. Specifically, we observed that anteriorly-oriented, non-optimal contacts also resulted in worse outcomes (i.e., slower, more variable movement profiles, opposing brain-behavior trajectories) compared to clinically-effective contacts tested in the current study. Thus, these data suggest that traditional clinical evaluations of therapeutic windows and associated side effects should be considered together with the neural and behavioral data reported herein to provide a more comprehensive outlook of optimal clinical programming strategies in the future.

To conclude, to our knowledge, this study was the first to establish the differential relationship between DBS-evoked cortical responses and quantitative movement profiles for indexing clinical outcomes related to optimal and non-optimal DBS programming settings (i.e., contact orientations) in PD. Specifically, we observed that longer-latency evoked cortical responses in the sensorimotor cortex ipsilateral to monopolar STN-DBS were largest when undergoing clinically-effective contact orientations, that finger tapping was smoother during optimal configurations, and that long-latency SM1 responses were differentially predictive of smoother movement profiles based on the direction of current administration. In addition, we report that the most common side effects observed regardless of stimulation protocol included muscle contractions, speech disturbances, and dizziness. However, spatially-specific side effects and dynamic changes in therapeutic windows (i.e., better or worse therapeutic settings) were induced by discrete changes in the direction in which current was administered (e.g., laterally- vs. anteriorly-oriented current spread, respectively). Importantly, these trajectories were consistent with worse outcomes observed in our neurophysiological and behavioral data induced by non-optimal stimulation settings, providing additional support for these metrics identifying potentially relevant optimal/non-optimal DBS programming strategies.

While our results are promising, the study is not without its limitations. First, it is typical for combined MEG-DBS studies to switch to effective bipolar stimulation settings from clinically-used monopolar ones, as this may help further reduce the artifact induced by DBS hardware (i.e., generator and cables)^35^. However, because the goal of the study was to identify the neurophysiological and behavioral correlates (and importantly, their interaction) of clinical outcomes relating to clinically-effective and non-effective contact orientations, we opted to retain monopolar stimulation settings during MEG to adhere more closely to the standard monopolar review of finger tapping performance. Although we applied the recommended methods for DBS artifact cleaning for source-level analyses (i.e., tSSS)^35^, we were limited in our evaluation of shorter latency responses (e.g., <2 ms), which may still be contaminated by the DBS artifact. Similarly, in agreement with previous electrophysiological studies of DBS parameter settings^12,13,16,20^, our experimental design administered low-frequency DBS (i.e., 6 Hz) during MEG to allow for sufficient time between each DBS pulse to precisely quantify disparate response latencies following DBS pulse onset, albeit low-frequency STN-DBS is not typically effective in eliciting clinical benefits for parkinsonian symptoms. Essentially, our relation of low-frequency neural activation to high-frequency (i.e., 130 Hz) clinical outcomes was motivated by prior studies in this area demonstrating similar DBS-evoked response profiles and/or clinical outcomes (e.g., bradykinesia) regardless of the frequency administered, which we presume would also be similar in our cohort^13,39^. Thus, our results demonstrate the neurophysiological correlates of clinical benefits relating to optimal and non-optimal contact orientations in particular, as opposed to delineating the precise mechanism of action of high-frequency DBS in isolation. In addition, future studies will undoubtedly benefit from the evaluation of numerous parameters of movement in parallel (e.g., standardized UPDRS III finger tapping, pronation-supination hand movements, flexion-extension hand movements, etc.), to elucidate the full extent of stimulation-evoked cortical responses for indexing more general decrements in motor function. Likewise, while the current study focused on DBS-evoked cortical responses as proxies for DBS clinical efficacy, future analyses of additional neurophysiological metrics that may prove informative for DBS programming (e.g., elevated beta synchronization in the STN)^40–43^ will be essential to provide a more comprehensive overview of the neurophysiological correlates of DBS clinical outcomes. Moreover, future investigations may also benefit from quantifying these neurophysiological features using more readily accessible or cost effective alternatives to MEG such as EEG or even newly available wearable MEG systems (i.e., optically pumped magnetometers)^44^ in order to advance the utilization of such markers in a clinical setting. Nevertheless, our data suggest that the brain-behavior dynamics assessed in the current study may serve as effective targets for optimizing DBS settings in patients with Parkinson’s disease. The development of such non-invasive and quantitative markers for DBS programming is of utmost importance, as it improves upon the current standards using subjective designation of therapeutic effects which largely demonstrate poor reliability and reproducibility^45,46^, and also, may ultimately increase the efficiency of clinical programming appointments for patients in the future.

## Methods

### Patient demographics

Twenty-four patients with Parkinson’s disease (*M*_age_ = 63.96 years old, 43–80 years old, 3 females) implanted with STN-DBS (Abbott Infinity DBS System, lead model: 6172, electrode: 6671, Abbott, Plano, Texas, USA) were recruited for this study from the Center for Movement Disorders and Neuromodulation at the University Hospital Düsseldorf. Exclusionary criteria included any medical illness affecting CNS function, any neurological or psychiatric disorder (except PD), severe depression (Beck Depression Inventory >30), or cognitive impairment (Mini-Mental State Examination <26). Patients were recorded in the clinically-defined medication OFF state following withdrawal of dopaminergic medication 12 h prior to study completion. For a comprehensive description of PD-relevant clinical information, see Table 1. Based on previous studies from our laboratory and others evaluating the neurophysiological correlates of DBS therapy in PD patients^12,13,16,20,47–49^, concomitant with effect size and confidence interval calculations based on relevant test statistics for each reported result (see Supplementary Tables 2–10), we derived that 20 participants would provide adequate power for all behavioral and neural analyses.

### Ethics approval

The local ethics committee at Heinrich-Heine University Düsseldorf approved the study (No. 2019-626_2) and all patients provided written informed consent in accordance with the Declaration of Helsinki.

### Monopolar review and accelerometer analysis of behavior

Participants were instructed to complete a finger tapping paradigm (item 3.4 of UDPRS Part III Examination) of ~10 consecutive tapping sequences as largely, quickly and precisely as possible with the thumb and index finger in the air and a triaxial accelerometer (ADXL335 iMEMS Accelerometer, Analog Devices Inc., Norwood, MA, USA)^50^ attached to the right index finger. Simultaneously, we applied monopolar DBS of the left STN at each directional and ring-shaped contact orientation (i.e., A-, B-, C-, omni-directional contacts) at therapeutically beneficial settings (i.e., 130 Hz, 60 µs pulse width, current therapeutic contact height, ≥clinically-effective stimulation amplitude). During testing, clinical improvement and immediate, sustained side effects were assessed. Of note, as monopolar review sessions use MDS-UPDRS recommendations for motor assessments to evaluate clinical improvement and sustained side effects (e.g., Item 3.4), we opted to adhere our paradigm to the current clinical standards for finger tapping assessments to ensure comparability with standardized movement protocols. To visualize the correspondence between the quantitative kinematic features and traditional clinical evaluations of motor impairment reported herein, see Supplementary Fig. 2.

In order to quantify finger tapping metrics on the single-trial level, we developed an event detection algorithm using custom-written scripts in MATLAB (Version 2021a). Finger tapping blocks were epoched (i.e., ~10 consecutive taps per contact orientation tested) and pre-processed. The acceleration signal was visually inspected for artifacts and filtered using a third order high-pass Butterworth filter (1 Hz cut-off frequency). Next, probable finger tapping events were detected at the single-trial level using a two-stage approach. First, probable tapping events were identified using a fixed-threshold algorithm based on the magnitude and jerk (i.e., rate of change of acceleration) percentile thresholds of the accelerometer vector (i.e., 90 and 95th percentiles, respectively). The resulting time windows of probable finger taps (i.e., time of movement onset to offset) were further confirmed using the *findpeaks* function in MATLAB (i.e., minimum peak prominence ≥2.5 SD above the accelerometer vector magnitude; minimum peak distance ≥100 ms). The resulting confirmed finger tapping events (i.e., movement onset to offset) were then used to quantify single-trial and grand-averaged behavioral metrics including normalized general acceleration magnitude in m/s^2^ (normed to tap duration; i.e., movement onset to offset in ms), inter-tap interval or tapping frequency (i.e., onset to onset distance in taps per second), and the coefficient of variation of each variable, reflecting tapping consistency in each metric across the finger tapping block.

### MEG data acquisition and coregistration with structural MRI

All recordings were performed in a three-layer magnetically-shielded room. With an acquisition bandwidth of 0.1–1660 Hz, neuromagnetic responses were sampled continuously at 5 kHz using a MEGIN/Elekta MEG system (MEGIN, Helsinki, Finland) with 306 magnetic sensors, including 204 planar gradiometers and 102 magnetometers. During MEG recordings, patients were instructed to rest with their eyes open and fixated on a crosshair while 6 Hz monopolar stimulation of the left STN was administered, so that the inter-pulse interval could be analyzed. Clinically-effective stimulation settings were applied (same as monopolar review above with exception of stimulation frequency) while ~240 pulses were collected for each contact orientation (i.e., A-, B-, C-, omni-directional). Prior to MEG acquisition, four coils were attached to the subject’s head and localized, together with fiducial and ~150 scalp surface points, using a three‐dimensional (3D) digitizer (FASTRAK 3SF0002, Polhemus Navigator Sciences, Colchester, Vermont). Throughout data acquisition, participants were monitored using a real-time audio-video feed from inside the magnetically-shielded room. MEG data from each patient were subjected to noise reduction using the signal space separation method with a temporal extension^51^. Only data from the gradiometers were used for further analysis. Each participant’s MEG data were coregistered with their pre-surgical structural T1-weighted MRI data prior to imaging analyses using an iterative closest-point rigid-body registration in Brainstorm^52^. These fits were manually corrected following visual inspection when appropriate. Structural MRI data were segmented and cortical surfaces were computed using the CAT12 toolbox in SPM^53^ using default setting and imported into Brainstorm. Individual cortical surfaces were down-sampled to 15,000 vertices for MEG source imaging analyses.

### MEG pre-processing and sensor-level analyses

Cardiac and ocular artifacts were removed from the data using signal-space projection (SSP) and the projection operator was accounted for during source reconstruction^54^. Epochs were of 200 ms duration, with 0 ms defined as the onset of the DBS pulse and the baseline being the −50 to 0 ms window. Epochs containing artifacts were rejected based on a fixed-threshold method of trial-wise neural amplitude (fT) and gradient (fT/cm/s) values exceeding 3 median absolute deviations, supplemented with visual inspection. On average, 216 ± 24 trials per patient and experimental run were used for further analysis. Importantly, the number of trials did not significantly differ as a function of experimental run (i.e., contact orientation A, B, C, omni; *F*(75) = 0.68, *p* = 0.564).

Artifact-free epochs per patient and experimental run were averaged with respect to the onset of the DBS pulse for each sensor in the array, and the resulting mean time series per sensor and patient were examined statistically to determine the specific time windows used for subsequent source analyses. We used a two-stage approach that included paired-sample *t*-tests against baseline across subjects, followed up with non-parametric permutation testing to control for multiple comparisons (initial threshold: *p* < 0.05, permutations: 10,000)^55,56^. The permutation procedure used Monte Carlo random sampling to estimate the empirical distribution of the t-statistic at each sensor and time point in the experimental epoch^55,56^. The resulting phase-locked, time-domain period that significantly differed from baseline (FDR-corrected at *p* < 0.005 and minimum duration of 5 ms for time and sensors) was used to guide subsequent time-domain source-level analyses to select the time window of interest. Further details of this method can be found in recent papers^57–60^.

### MEG source imaging

Source images were computed using unconstrained weighted minimum norm estimation (MNE) using the default parameters in Brainstorm^52^. The noise covariance matrix was obtained from 2 min of empty room recording acquired on the day of each patient’s measurement. Of note, the same MEG pre-processing steps (i.e., tSSS, notch filter) were applied to the empty room recordings. The resulting whole-brain maps were 4-dimensional estimates of current density per vertex, per time sample from −50 to 150 ms locked to DBS pulse onset averaged across all trials. These data were *z*-score normalized to the baseline (−50 to 0 ms). Using the temporal clusters identified in the sensor-level analysis, these maps were grand-averaged using the dominant orientation (i.e., greatest amplitude increase/decrease from baseline) over the significant 3–10 ms and 16–26 ms time window following the DBS pulse onset across all trials, experimental runs (i.e., contact orientations) and patients to determine the peak vertex of the time-domain neural response. From this peak, the average response amplitude (dominant orientation and norm across three orientations; *z*-scored time series was extracted per task condition across the pre-defined time window of interest to derive estimates of the time-domain response for each patient.

### Statistical analyses

First, in order to evaluate the influence of optimal and non-optimal STN-DBS current administration (i.e., contact orientation) on accelerometer metrics of fine hand movements and evoked cortical responses, linear mixed effects models (LMEs) of contact orientation (fixed effect factor with 5 levels—see below) controlling for acquisition order, total electrical energy delivered (TEED)^24^, and subject (random effect) on behavioral and neural outcomes were conducted separately using the *lme4* package in R (Version 4.0.3). Of note, TEED was included as a covariate of no interest in our LMEs in order to control for the quantifiable differences in the volume of tissue activated based on the inclusion of both directional and omni-directional contact configurations in our statistical analysis. Additionally, because we aimed to systematically evaluate one parameter space of DBS programming (i.e., contact orientations) on neural and behavioral correlates of clinical outcomes, all other parameter settings were held constant by using the patient’s clinically-effective parameter settings at the time of study enrollment (i.e., amplitude, pulse width, frequency). This resulted in patient-specific variations in stimulation amplitude, which was controlled for in our group-level analyses by including TEED as a covariate of no interest in relevant models. For a summary of patient-specific parameter settings used in the current study, see Supplementary Table 1.

**Fig. 5:**
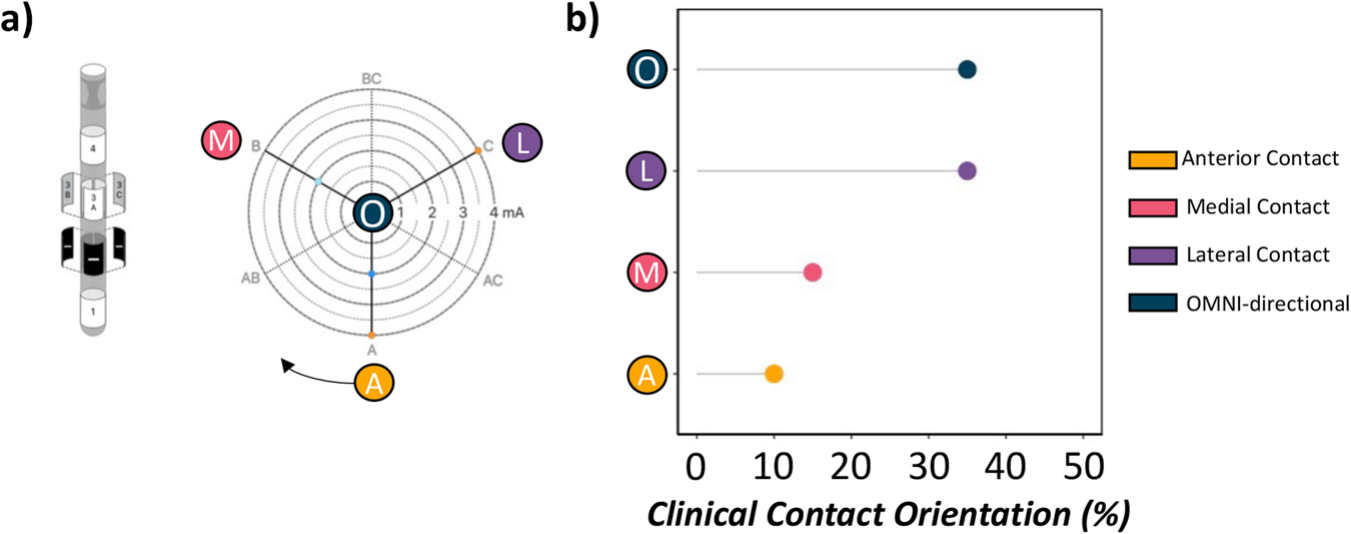
STN-DBS contact orientations. **a** Schematic of Abbott Infinity DBS electrode with directional (i.e., anterior, medial, lateral) and ring-shaped contact designations (i.e., omni-directional) confirmed anatomically using patient x-rays, CT scans and surgical notes. **b** Proportion of sample’s clinically-effective contact orientation (in %) based on clinical settings at the time of participation. The greatest proportion of clinically-effective contact orientations were identified as omni-directional and laterally-oriented contacts (>76% of the sample).

Next, categorical definitions of optimal (i.e., subject-specific clinically-effective contact orientation or omni-directional stimulation based on pre-defined clinical settings) and non-optimal contact orientations were confirmed based on their anatomical orientation within the left STN (i.e., anterior, medial, lateral, omni) using the patient’s x-rays, CT scans and surgical notes (Fig. 5 and Supplementary Fig. 1). For example, metallic demarcations on Abbott electrodes denoting the anatomical orientation of contact “A” about the DBS lead were used to confirm the orientation of arbitrarily defined A-, B- and C-directional contacts (see Supplementary Fig. 1). The resulting experimental runs with the patient’s pre-defined clinical contact would then be categorically assigned as their “clinical contact,” with the remaining three experimental runs assigned to their anatomical orientation within the left STN (i.e., anterior, medial, lateral, or omni-directional). For example, if a patient’s clinically-effective contact was oriented anteriorly, then all non-optimal experimental runs would be assigned according to the remaining medial and lateral directional contact orientations, as well as the omni-directional current administration. In contrast, if a patient’s optimal contact orientation was the omni-directional ring-shaped current administration, then each additional non-optimal experimental run would be assigned according to their directional anterior, medial or lateral anatomical orientations within the left STN. The resulting variable was factorized with 5 potential levels (i.e., clinical contact, anterior contact, medial contact, lateral contact, omni-directional). Importantly, all LME post-hoc analyses of trending and significant main effects were corrected for multiple comparisons using Tukey’s multiple comparison test.

Next, we aimed to evaluate the predictive capacity of DBS-evoked sensorimotor responses on behavioral outcomes assessed outside the scanner. To index relevant metrics of finger tapping movement profiles in the current sample, we conducted an exploratory factor analysis (EFA) to define a single component of movement using a compilation of accelerometer metrics exhibiting significant alterations as a function of contact orientation (i.e., general acceleration magnitude, acceleration variability, tapping frequency, tap frequency variability). We began with a set list of four measures and progressively removed individual variables based on poor loadings (*λ* < 0.70), and overall model fit. Criteria for good model fit included a non-statistically significant chi square, a root mean squared error approximation (RMSEA) < 0.06, a comparative fit index (CFI) > 0.95, and a standardized root mean squared residual (SRMR) < 0.08 based on standards in the literature^61^. The best fitting model was used to define a latent variable for which a movement profile score was extracted per participant. Modeling and component extraction was completed using *lavaan* and *principal* functions in R, respectively. As such, finger tapping movement profile scores were subsequently extracted per patient and entered as dependent variables in our LME of finger tapping movement profiles as a function of contact orientation, neural response amplitude and their interaction. Of note, lower finger tapping movement profiles scores are reflective of smoother finger tapping movements based on the directionality observed during the application of clinically-effective contact orientations (i.e., lower acceleration magnitude, lower acceleration variability, increased tapping frequency). Post-hoc analyses of LME interaction effects were probed using Fisher Z-transformations of each brain-behavioral effect to evaluate the differences in the predictive capacities of each effect as a function of contact orientation^62,63^. Finally, therapeutic windows and associated side effects for each optimal and non-optimal contact orientation are reported for the current sample as a descriptive comparison to the neurophysiological and behavioral data reported herein.

## Supplementary information

Supplementary Material

## Data Availability

The anonymized data from this study will be made available to investigators upon request from the corresponding authors.

## Code availability

The code used in this study will be made available to investigators upon request from the corresponding authors.
