# Supplementary material for: DBS-evoked cortical responses index optimal contact orientations and motor outcomes in Parkinson’s disease
Source: NPJ Parkinsons Dis. 2023 Mar 11;9:37. doi: 10.1038/s41531-023-00474-4 (PMC10008535; doi:10.1038/s41531-023-00474-4)
Supplement: Supplementary file 1 — Supplementary Material [file 41531_2023_474_MOESM1_ESM.pdf]

Supplementary Figures

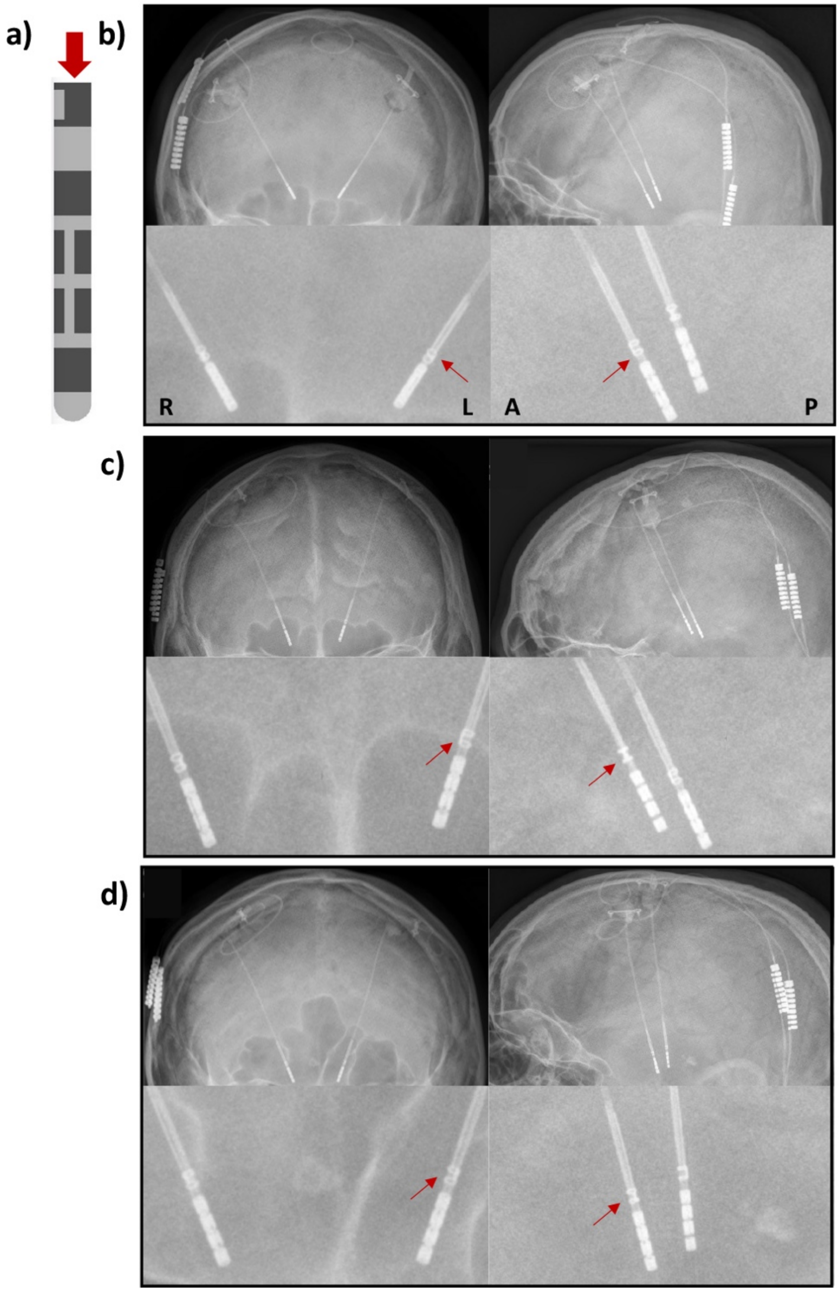

Figure S1. Anatomical Confirmation of Contact Orientation in Exemplary Subjects. Anatomical orientations of segmented contacts (i.e., A-, B- and C-directional contacts) from the left STN were confirmed anatomically by reviewing patient x-rays, CT scans and surgical notes. Panel a) Schematic of the Abbott electrode visualized using Lead-DBS denotes a metal demarcation (i.e., red arrow) producing a metallic artifact in the resulting post-operative x-rays. The metallic demarcation denotes the orientation of contact "A" within the DBS lead. Contact orientations reflecting an anteriorly-oriented contact A (panel b), a medially-oriented contact A (panel c) and a laterally-oriented contact A (panel d) for exemplary subjects are displayed. Red arrows denote this demarcation for the DBS electrode implanted in the left STN for coronal and sagittal displays per subject. R = right; L = left; A = anterior; P = posterior.

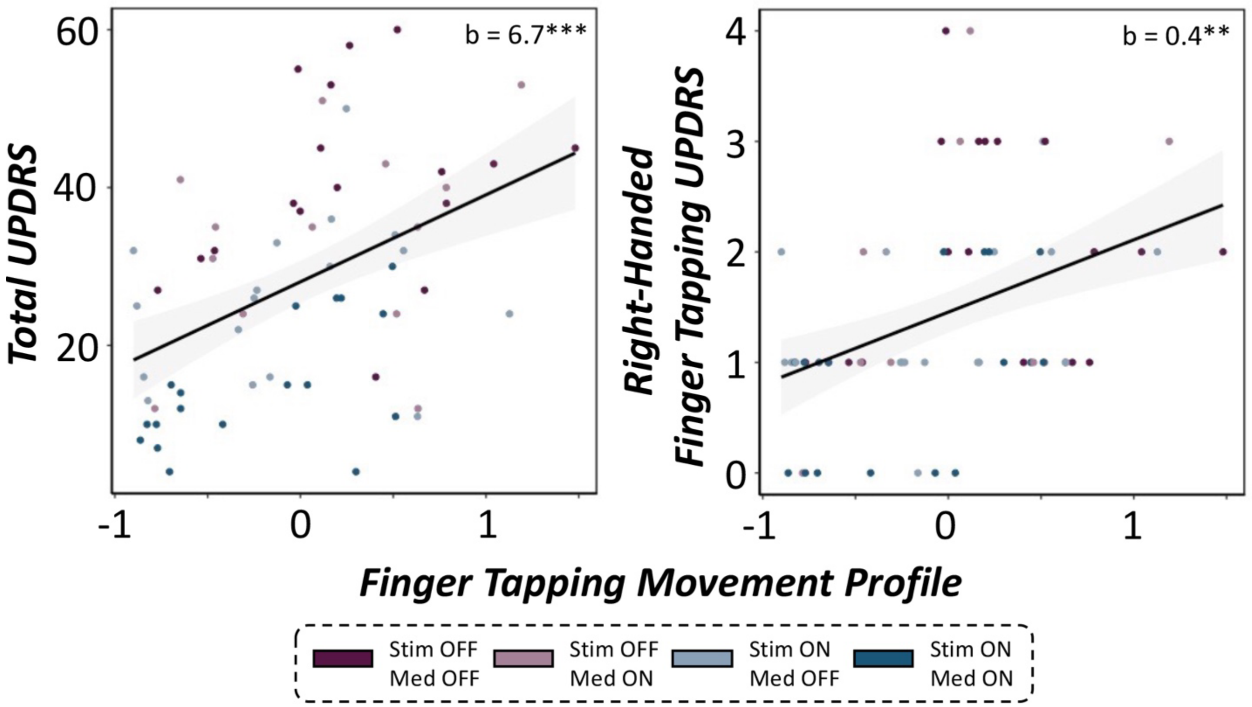

Figure S2. Quantitative Finger Tapping Movement Profiles Relate To Traditional Clinical Evaluations of Motor Impairment. Confirmatory factor analysis was used to derive a single finger tapping movement profile per patient (N = 20) in a supplementary dataset collected with traditional UPDRS-III testing conditions during changing medication and stimulation statuses. Factor scores were computed based on metrics described in the primary analysis (i.e., acceleration magnitude, acceleration variability, tapping frequency, see *Statistical analyses*), with lower values indicative of better movement profiles. Linear mixed effects models of finger tapping movement profiles on total UPDRS-III (left) and right-handed finger tapping (right) Item 3.4 UPDRS scores were conducted separately. Lower movement profile scores (i.e., better behavioral performance) were predictive of lower UPDRS total and subscores (i.e., less severe motor deficits) regardless of medication and stimulation status. Taken together, these data suggest that quantitatively-derived finger tapping movement profiles as measured in the current study relate well to traditional clinical evaluations of motor symptom severity. 95% confidence intervals are displayed in gray for each regression line. $**p < .005$, $***p < .001$.

Supplementary Tables

Table S1. DBS Parameter Settings for Behavioral and MEG Recordings

subID	Contact	Frequency (Hz)	Pulse Width (μs)	Amplitude (mA)
sub-001	A,B,C,OMNI	130, 6	60	4
sub-004	A,B,C,OMNI	130, 6	60	1
sub-005	A,B,C,OMNI	130, 6	60	2
sub-006	A,B,C,OMNI	130, 6	60	3
sub-007	A,B,C,OMNI	130, 6	60	2
sub-008	A,B,C,OMNI	130, 6	60	1
sub-010	A,B,C,OMNI	130, 6	60	2
sub-011	A,B,C,OMNI	130, 6	60	2
sub-012	A,B,C,OMNI	130, 6	60	1
sub-013	A,B,C,OMNI	130, 6	60	2
sub-014	A,B,C,OMNI	130, 6	60	2
sub-015	A,B,C,OMNI	130, 6	60	3
sub-016	A,B,C,OMNI	130, 6	60	2
sub-017	A,B,C,OMNI	130, 6	60	3
sub-018	A,B,C,OMNI	130, 6	60	2
sub-019	A,B,C,OMNI	130, 6	60	3
sub-020	A,B,C,OMNI	130, 6	60	3
sub-021	A,B,C,OMNI	130, 6	60	2
sub-024	A,B,C,OMNI	130, 6	60	4
sub-025	A,B,C,OMNI	130, 6	60	4

Note. Left STN DBS contact settings for monopolar review sessions (i.e., delivered at 130 Hz) and MEG recording sessions (i.e., delivered at 6 Hz).

Table S2. Model results of contact orientation on single-trial finger tapping acceleration magnitude during STN-DBS.

Acceleration Magnitude during STN-DBS					
**Effect**	**F**	**p**	**Effect Size (d)**	**Lower CI**	**Upper CI**
Contact Orientation	9.58	0.001*	0.19	**0.07**	**0.31**
Post Hoc Testing					
**Main Effect of Contact Orientation**	**t**	**p**	**Effect Size (d)**	**Lower CI**	**Upper CI**
Clinical vs. Anterior	-0.88	0.380	-0.03	-0.09	0.03
Clinical vs. Medial	-3.84	0.001*	-0.12	**-0.17**	**-0.06**
Clinical vs. Lateral	-4.82	0.001*	-0.14	**-0.20**	**-0.09**
Clinical vs. Omni	1.64	0.102	0.05	-0.01	0.11
Anterior vs. Medial	-2.45	0.014	-0.07	**-0.13**	**-0.01**
Anterior vs. Lateral	-3.52	0.001*	-0.11	**-0.16**	**-0.05**
Anterior vs. Omni	1.88	0.060	0.06	**0.00**	**0.12**
Medial vs. Lateral	-1.37	0.172	-0.04	-0.10	0.02
Medial vs. Omni	4.23	0.001*	0.13	**0.07**	**0.19**
Lateral vs. Omni	4.76	0.001*	0.14	**0.08**	**0.20**

Note. Cohen's d effect sizes and associated 95% confidence intervals (CI) were calculated based on appropriate test statistics using the *effectsize* package in R. Bolded values indicate statistical significance and trending effects based on $p_{corrected} < .05$ and .10, respectively following Tukey's correction for multiple comparisons and non-overlapping confidence intervals of effect sizes. *$p_{corrected} < .001$.

Table S3. Model results of contact orientation on single-trial finger tapping acceleration magnitude variability during STN-DBS.

Coefficient of Variation in Acceleration Magnitude					
**Effect**	**F**	**p**	**Effect Size (d)**	**Lower CI**	**Upper CI**
Contact Orientation	19.62	0.001*	0.27	**0.15**	**0.38**
Post Hoc Testing					
**Main Effect of Contact Orientation**	**t**	**p**	**Effect Size (d)**	**Lower CI**	**Upper CI**
Clinical vs. Anterior	-4.70	0.001*	-0.14	**-0.20**	**-0.08**
Clinical vs. Medial	1.07	0.284	0.03	-0.03	0.09
Clinical vs. Lateral	-2.08	0.038	-0.06	**-0.12**	**0.00**
Clinical vs. Omni	-6.06	0.001*	-0.18	**-0.24**	**-0.12**
Anterior vs. Medial	5.65	0.001*	0.17	**0.11**	**0.23**
Anterior vs. Lateral	2.50	0.012	0.07	**0.02**	**0.13**
Anterior vs. Omni	-1.21	0.225	-0.04	-0.09	0.02
Medial vs. Lateral	-2.96	0.003	-0.09	**-0.15**	**-0.03**
Medial vs. Omni	-6.13	0.001*	-0.18	**-0.24**	**-0.12**
Lateral vs. Omni	-3.26	0.001*	-0.10	**-0.16**	**-0.04**

Note. Cohen's d effect sizes and associated 95% confidence intervals (CI) were calculated based on appropriate test statistics using the *effectsize* package in R. Bolded values indicate statistical significance and trending effects based on $p_{corrected} < .05$ and .10, respectively following Tukey's correction for multiple comparisons and non-overlapping confidence intervals of effect sizes. *$p_{corrected} < .001$.

Table S4. Model results of contact orientation on single-trial tapping frequency during STN-DBS.

Tap Frequency					
**Effect**	**F**	**p**	**Effect Size (d)**	**Lower CI**	**Upper CI**
Contact Orientation	2.42	0.047	0.14	**0.02**	**0.26**
Post Hoc Testing					
**Main Effect of Contact Orientation**	**t**	**p**	**Effect Size (d)**	**Lower CI**	**Upper CI**
Clinical vs. Anterior	2.03	0.042	0.06	**0.00**	**0.12**
Clinical vs. Medial	-0.93	0.355	-0.03	-0.09	0.03
Clinical vs. Lateral	0.18	0.853	0.01	-0.05	0.07
Clinical vs. Omni	0.92	0.360	0.03	-0.03	0.09
Anterior vs. Medial	-2.85	0.004	-0.09	**-0.15**	**-0.03**
Anterior vs. Lateral	-1.73	0.084	-0.05	**-0.11**	**0.01**
Anterior vs. Omni	-0.78	0.434	-0.02	-0.08	0.04
Medial vs. Lateral	0.99	0.323	0.03	-0.03	0.09
Medial vs. Omni	1.48	0.140	0.05	-0.01	0.11
Lateral vs. Omni	0.58	0.559	0.02	-0.04	0.08

Note. Cohen's d effect sizes and associated 95% confidence intervals (CI) were calculated based on appropriate test statistics using the *effectsize* package in R. Bolded values indicate statistical significance and trending effects based on $p_{corrected} < .05$ and .10, respectively following Tukey's correction for multiple comparisons and non-overlapping confidence intervals of effect sizes.

Table S5. Model results of contact orientation on single-trial tap frequency variability during STN-DBS.

Coefficient of Variation in Tap Frequency					
**Effect**	**F**	**p**	**Effect Size (d)**	**Lower CI**	**Upper CI**
Contact Orientation	5.32	0.001*	0.14	**0.02**	**0.26**
Post Hoc Testing					
**Main Effect of Contact Orientation**	**t**	**p**	**Effect Size (d)**	**Lower CI**	**Upper CI**
Clinical vs. Anterior	-1.71	0.087	0.05	**-0.01**	**0.11**
Clinical vs. Medial	3.89	0.001*	0.12	**0.06**	**0.18**
Clinical vs. Lateral	2.95	0.003	0.09	**0.03**	**0.15**
Clinical vs. Omni	-0.94	0.349	-0.03	-0.09	0.03
Anterior vs. Medial	1.67	0.096	0.05	**-0.01**	**0.11**
Anterior vs. Lateral	1.06	0.291	0.03	-0.03	0.09
Anterior vs. Omni	-1.95	0.051	-0.06	**-0.12**	**0.00**
Medial vs. Lateral	-0.53	0.595	-0.02	-0.08	0.04
Medial vs. Omni	-3.66	0.001*	-0.11	**-0.17**	**-0.05**
Lateral vs. Omni	-2.86	0.004	-0.09	**-0.15**	**-0.03**

Note. Cohen's d effect sizes and associated 95% confidence intervals (CI) were calculated based on appropriate test statistics using the *effectsize* package in R. Bolded values indicate statistical significance and trending effects based on $p_{corrected} < .05$ and .10, respectively following Tukey's correction for multiple comparisons and non-overlapping confidence intervals of effect sizes. *$p_{corrected} < .001$.

Table S6. Model results of contact orientation on SM1 medium-latency evoked response amplitude during STN-DBS.

SM1 Medium-Latency Evoked Response Amplitude					
**Term**	**F**	**p**	**Effect Size (d)**	**Lower CI**	**Upper CI**
Contact Orientation	0.28	0.892	0.12	-0.32	0.56

Note. Cohen's d effect sizes and associated 95% confidence intervals (CI) were calculated based on appropriate test statistics using the *effectsize* package in R. Bolded values indicate statistical significance and trending effects based on $p_{corrected} < .05$ and .10, respectively following Tukey's correction for multiple comparisons and non-overlapping confidence intervals of effect sizes.

Table S7. Model results of contact orientation on SM1 medium-latency evoked response latency during STN-DBS.

SM1 Medium-Latency Evoked Response Latency					
**Term**	**F**	**p**	**Effect Size (d)**	**Lower CI**	**Upper Ci**
Contact Orientation	0.49	0.744	0.18	-0.32	0.68

Note. Cohen's d effect sizes and associated 95% confidence intervals (CI) were calculated based on appropriate test statistics using the *effectsize* package in R. Bolded values indicate statistical significance and trending effects based on $p_{corrected}$ < .05 and .10, respectively following Tukey's correction for multiple comparisons and non-overlapping confidence intervals of effect sizes.

Table S8. Model results of contact orientation on SM1 long-latency evoked response amplitude during STN-DBS.

SM1 Long-Latency Evoked Response Amplitude					
**Effect**	**F**	**p**	**Effect Size (d)**	**Lower CI**	**Upper CI**
Contact Orientation	2.40	0.058	0.37	**-0.10**	**0.85**
Post Hoc Testing					
**Main Effect of Contact Orientation**	**t**	**p**	**Effect Size (d)**	**Lower CI**	**Upper CI**
Clinical vs. Anterior	0.93	0.357	0.11	-0.13	0.35
Clinical vs. Medial	1.09	0.279	0.13	-0.11	0.37
Clinical vs. Lateral	2.48	0.016	0.30	**0.06**	**0.54**
Clinical vs. Omni	2.55	0.013	0.31	**0.06**	**0.55**
Anterior vs. Medial	0.08	0.940	0.01	-0.23	0.25
Anterior vs. Lateral	1.44	0.154	0.17	-0.06	0.41
Anterior vs. Omni	1.48	0.143	0.18	-0.06	0.42
Medial vs. Lateral	1.46	0.148	0.18	-0.06	0.41
Medial vs. Omni	1.51	0.136	0.18	-0.06	0.42
Lateral vs. Omni	0.01	0.993	0.00	-0.23	0.22

Note. Cohen's d effect sizes and associated 95% confidence intervals (CI) were calculated based on appropriate test statistics using the *effectsize* package in R. Bolded values indicate statistical significance and trending effects based on $p_{corrected} < .05$ and .10, respectively following Tukey's correction for multiple comparisons and non-overlapping confidence intervals of effect sizes.

Table S9. Model results of contact orientation on SM1 long-latency evoked response latency during STN-DBS.

SM1 Long-Latency Evoked Response Latency					
**Term**	**F**	**p**	**Effect Size (d)**	**Lower CI**	**Upper CI**
Contact Orientation	1.30	0.279	0.30	-0.22	0.82

Note. Cohen's d effect sizes and associated 95% confidence intervals (CI) were calculated based on appropriate test statistics using the *effectsize* package in R. Bolded values indicate statistical significance and trending effects based on $p_{corrected}$ < .05 and .10, respectively following Tukey's correction for multiple comparisons and non-overlapping confidence intervals of effect sizes.

Table S10. Model results of contact orientation, SM1 long-latency evoked response amplitude and their interaction on finger tapping movement profiles during STN-DBS.

Finger Tapping Movement Profile Score					
**Effect**	**F**	**p**	**Effect Size (d)**	**Lower CI**	**Upper CI**
Evoked Response Amplitude	0.25	0.622	0.16	-0.47	0.78
Contact Orientation	5.03	0.002	0.78	**0.07**	**1.48**
Interaction	4.35	0.006	0.73	**0.02**	**1.43**
Post Hoc Testing					
**Main Effect of Contact Orientation**	**t**	**p**	**Effect Size (d)**	**Lower CI**	**Upper CI**
Clinical vs. Anterior	1.65	0.108	0.20	-0.04	0.44
Clinical vs. Medial	0.07	0.946	0.01	-0.23	0.24
Clinical vs. Lateral	1.22	0.230	0.15	-0.09	0.38
Clinical vs. Omni	-0.84	0.406	-0.10	-0.34	0.14
Anterior vs. Medial	-1.43	0.162	-0.17	-0.41	0.07
Anterior vs. Lateral	0.33	0.742	0.04	-0.20	0.28
Anterior vs. Omni	-1.75	0.089	-0.21	**-0.45**	**0.03**
Medial vs. Lateral	1.23	0.228	0.15	-0.09	0.38
Medial vs. Omni	-0.79	0.433	-0.10	-0.33	0.14
Lateral vs. Omni	-1.55	0.131	-0.19	-0.42	0.05
**Interaction**	**Z**	**p**	**Effect Size (r)**	**Lower CI**	**Upper CI**
Clinical vs. Anterior	2.325	0.018	-0.56	**-0.78**	**-0.10**
Clinical vs. Medial	-0.80	0.424	-0.23	-0.62	0.32
Clinical vs. Lateral	-0.40	0.689	-0.56	-0.56	0.41
Clinical vs. Omni	0.22	0.826	0.06	-0.45	0.53
Anterior vs. Medial	1.22	0.222	0.33	-0.21	0.68
Anterior vs. Lateral	1.47	0.142	0.39	-0.14	0.70
Anterior vs. Omni	2.60	0.009	0.60	**0.18**	**0.80**
Medial vs. Lateral	0.31	0.757	0.09	-0.43	0.55
Medial vs. Omni	1.01	0.312	0.28	-0.26	0.65
Lateral vs. Omni	0.58	0.562	0.17	-0.37	0.59

Note. Cohen's d effect sizes and associated 95% confidence intervals (CI) were calculated based on appropriate test statistics using the *effectsize* package in R. Bolded values indicate statistical significance and trending effects based on $p_{corrected}$ < .05 and .10, respectively following Tukey's correction for multiple comparisons and non-overlapping confidence intervals of effect sizes.
